# Appropriate shape of a stylet for tracheal intubation using the McGrath® MAC videolaryngoscope in neonates: a randomized crossover simulation study

**DOI:** 10.1186/s40981-025-00772-1

**Published:** 2025-02-17

**Authors:** Katsuhide Masui, Naoyuki Tsunoda, Ayaka Ito, Takashi Asai

**Affiliations:** Department of Anesthesiology, Dokkyo Medical University Saitama Medical Center, 2-1- 50 Minami-Koshigaya, Koshigaya, Saitama 343-8555 Japan

**Keywords:** Neonates, Tracheal intubation, Videolaryngoscope, Stylet

## Abstract

**Background:**

Guidelines on airway management in neonates and infants recommend using a stylet when a videolaryngoscope is used, but it is not clear if the use of a stylet facilitates tracheal intubation and which shape of the stylet is suitable in neonates.

**Methods:**

As a preliminary simulation study of a randomized controlled cross-over design, 25 anesthesiologists (3 specialists, 11 senior residents, and 11 junior residents) used a McGrath® MAC videolaryngoscope (Covidien, Medtronic, Tokyo, Japan) blade 1 for tracheal intubation (of a 3.5-mm ID Shiley™ tube with a cuff), with one of four differently shaped stylets (C-shaped, J-shaped, hockey stick-shaped and double C-shaped) or without a stylet in a manikin of a neonate, and compared intubation times.

**Results:**

Compared with intubation time without the use of a stylet, intubation time was significantly longer with the use of the J-shaped stylet (*P* = 0.007; median (95% CI) difference: 2 (1 to 2) s) or with the hockey stick-shaped stylet (*P* = 0.0002; median (95% CI) difference: 9 (9 to 10) s). In contrast, intubation time was similar between no stylet and the C-shaped stylet (*P* = 0.90; median (95% CI) difference: 0 (0 to 0) s) or between no stylet and the double C-shaped style (*P* = 0.60; median (95% CI) difference: 0 (0 to 0) s).

**Conclusions:**

In conclusion, while time to tracheal intubation would be similar with and without the use of a stylet, the shape of the stylet would affect intubation time in neonates.

## Introduction

Videolaryngoscopes are useful for tracheal intubation in adults and in children, with and without difficult airways [1–4]. Guidelines on airway management recommend using a videolaryngoscope in adults with difficult airways [1, 2] and to use a videolaryngoscope as first choice in neonates and infants [3].

One possible cause of difficulty in tracheal intubation with a videolaryngoscope (particularly with a hyperangulated blade) is that, even when the glottis is clearly seen on the videoscreen, it may frequently be difficult to drive the tip of a tracheal tube toward the glottis [3, 4], and thus the use of a stylet is usually required. Several differently shaped stylets have been proposed, but some studies have indicated that the shape of the stylet may affect the ease of tracheal intubation in adults [5–8].

Guidelines on airway management in neonates and infants also recommend using a stylet when a hyperangulated videolaryngoscope blade is used [3], because neonates and infants are at increased risk of hypoxia and airway obstruction if tracheal intubation is difficult. Nevertheless, there has been only one study which compared the efficacy of two differently shaped stylets in infants [9], with another one in children [10]. Because there may be structural differences of the upper airways between neonates, infants, children and adults [11], it is not clear if the use of a stylet facilitates tracheal intubation and which shape of the stylet is suitable for tracheal intubation using a videolaryngoscope in neonates.

The purpose of this preliminary simulation study was to assess if the use of a stylet facilitates tracheal intubation and if the shape of the stylet affects the ease of tracheal intubation using a videolaryngoscope in neonates.

## Methods

We were planning to compare the use and non-use of a stylet and compare four differently shaped stylets in the ease of tracheal intubation in neonates, but we felt that it would be more ethical to carry out first, a preliminary study in a manikin of a neonate, because the efficiency of some shapes of stylets were predicted to be less effective. The study protocol was submitted to the research ethics committee which informed us that the study did not require a formal review, because this study was not a study on human subjects. Written informed consent was obtained from all the participants.

**Fig. 1:**
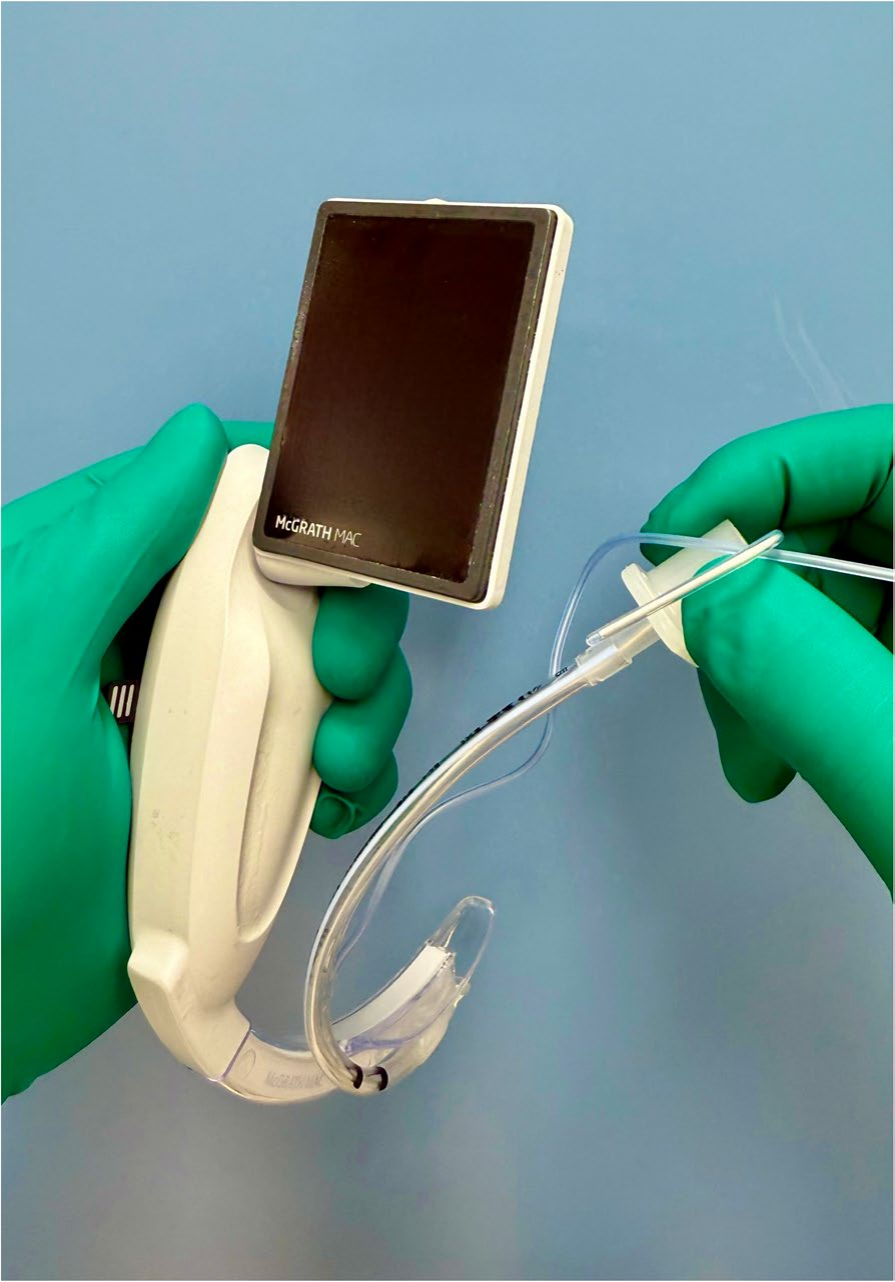
Double C-shaped stylet. The stylet has the distal part of the tracheal tube bent along the shape of the blade, with a 45-degree bend at the connection between the blade and handle of the McGrath® MAC videolaryngoscope

As a randomized controlled cross-over design, the use of four differently shaped stylets were compared with no stylet in the ease of tracheal intubation in a manikin of a neonate (Laerdal® Medical, Stavanger, Norway). Twenty-five anesthesiologists (3 specialists, 11 senior residents, and 11 junior residents) with at least 1 year of experience in pediatric anesthesia used a McGrath® MAC videolaryngoscope (Covidien, Medtronic, Tokyo, Japan) blade 1 for tracheal intubation, with one of four differently shaped stylets (C-shaped, J-shaped, hockey stick-shaped, and double C-shaped) or without a stylet. The shape of stylet (tracheal intubation stylet (2.0-mm OD, 255 mm), Smith Medical, Minneapolis, USA) is defined as follows: the C-shaped stylet follows the curve of the laryngoscope blade so that the curve looked like the letter C, the J-shaped stylet has its tip formed into the letter J, the hockey stick-shaped stylet has the tip bent at a 90-degree angle, and the double C-shaped stylet (Fig. 1) has the distal part of the tracheal tube bent along the shape of the blade, with a 45-degree bend at the connection between the blade and handle of the McGrath® MAC [12]. A 3.5-mm ID tracheal tube with a cuff (Shiley™ oral/nasal endotracheal tube with intermediate cuff, Covidien, Mansfield, USA) was used for all the intubation attempts, and a water-soluble lubricant was applied to its cuff before each use. The order of intubation methods was randomized.

The primary outcome measure was time to tracheal intubation on the first attempt, and the secondary outcome measure was the success rate of tracheal intubation at the first attempt. The time to tracheal intubation was defined as the time from when the tip of the blade entered the oral cavity until the anesthesiologist declared that the tube was inserted to the trachea. One of the investigators (KM) assessed the position of the tracheal tube, and if the tube was not inserted in the trachea, it was judged failure. We also judged that tracheal intubation failed, if it took more than 45 s [13]. This cut-off valve was based on our previous study of children under 2 years age, in which the median time to tracheal intubation using a McGrath blade 1 and a C-shaped stylet was 45 s. If tracheal intubation failed at the first attempt, another attempt was allowed.

Friedman’s two-way analysis of variance was used to compare the time to intubation between five different methods, and if this indicated a significant difference, Wilcoxon rank sum test was used to compare the intubation time between no use of a stylet and each of differently shaped stylet. *P* < 0.05 was considered as significant. 95% confidence intervals for median difference, with and without the use of a differently shaped stylet, were also calculated.

Power analysis was carried out for the primary outcome measure (time to tracheal intubation on the first attempt). In our previous studies of children in whom the McGrath® MAC blade 1 and a C-shaped stylet was used, the mean time (and the standard deviation) was 38 (7.4) s [13] and 40 (12.9) s [14]. From these studies, we estimated the mean time with a C-shaped stylet would be 40 s, with the standard deviation of 12 s. We considered that the difference of 20% (that is 8 s) in intubation time would be minimally clinically meaningful. To detect this difference, with a power of 0.8, and *P* = 0.05, the minimum of 21 participants would be required. To obtain a reasonably accurate estimation of the difference between groups, we decided to study 25 participants.

## Results

**Fig. 2:**
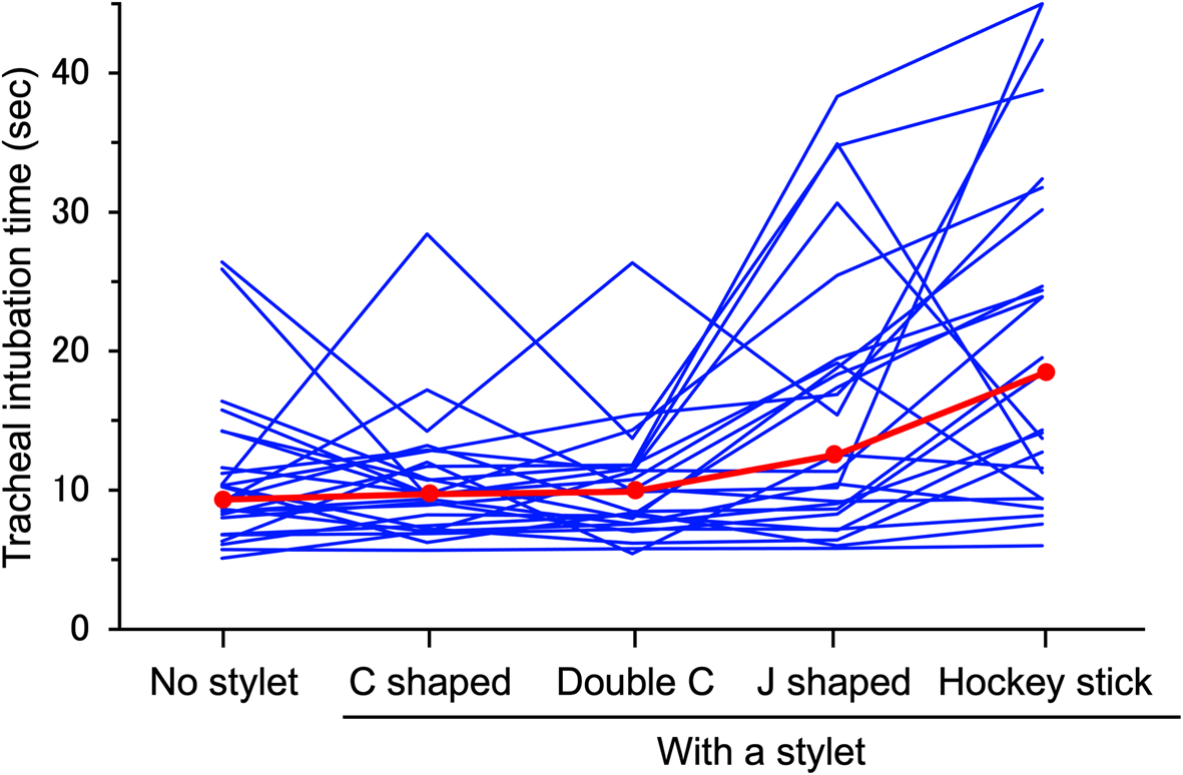
Individual data (blue lines) and the medians (red lines) for time to tracheal intubation (sec), with no style, or with one of four differently shaped stylet

Tracheal intubation was always successful with and without the use of stylet, expect for the hockey stick-shaped stylet (Table 1). Friedman’s two-way analysis of variance indicated a significant difference in the time to intubation between five different methods (*P* = 0.028) (Fig. 2). Compared with intubation time without the use of a stylet, intubation time was significantly longer with the use of the J-shaped stylet (*P* = 0.007; median (95% CI) difference: 2 (1 to 2) s) or with the hockey stick-shaped stylet (*P* = 0.0002; median (95% CI) difference: 9 (9 to 10) s). In contrast, intubation time was similar between no stylet and the C-shaped stylet (*P* = 0.90; median (95% CI) difference: 0 (0 to 0) s) or between no stylet and the double C-shaped style (*P* = 0.60; median (95% CI) difference: 0 (0 to 0) s).

**Table 1:** Success rates, time to tracheal intubation (median [interquartile range]), and 95% confidence intervals for median difference in intubation time with and without the use of a stylet

Type of stylet	Successful intubation at the first attempt	Successful intubation within two attempts	Time to tracheal intubation (s)	Median (95% CI) difference (from no stylet) (s)
No stylet	25 (100%)	―	9.3 [8.0–11.6]	―
C-shaped	25 (100%)	―	9.7 [7.4–12.0]	0 [0, 0]
Double C-shaped	25 (100%)	―	9.9 [7.6–11.6]	0 [0, 0]
J-shaped	25 (100%)	―	12.6 [8.7–19.1]	2 [1, 2]
Hockey stick-shaped	23 (92%)	25 (100%)	18.4 [11.3–30.2]	9 [9, 10]

## Discussion

From this preliminary simulation study, we have found that, in neonates, the use of a stylet may not facilitate tracheal intubation and that the shape of the stylet may significantly affect the ease of tracheal intubation using a videolaryngoscope.

When a J-shaped or a hockey stick-shaped stylet was used, the tip of a tracheal tube frequently impacted on the inner wall of the oral cavity, or the tip of the tube was also frequently not visible on the videoscreen of the McGrath® MAC. In addition, even if the tip of the tube was visible on the videoscreen, it was frequently difficult to drive the tube to the trachea. These results are consistent with a study of infants in whom tracheal intubation using a C-MAC® videolaryngoscope was attempted [9]; intubation time was significantly longer with the use of a hockey stick-shaped stylet than a C-shaped stylet.

In adults, it is known that the use of an introducer (such as a stylet and a gum-elastic bougie) facilitates tracheal intubation when a videolaryngoscope is used. In contrast, in our study of a neonate model, there was no difference in intubation time with and without the use of a C-shaped or a double C-shaped stylet (Table 1, Fig. 2). The exact reason for this difference is not clear, but structural differences of the upper airways between infants and adults might be the reason [11]. This possibility is partly supported by the fact that, in infants, intubation times are similar with and without the use of a stylet when a conventional direct laryngoscope is used [15]. From these results, it may be assumed that, when McGrath® MAC videolaryngoscope is used in anesthetized neonates, tracheal intubation would be achieved smoothly without the use of a stylet or with the use of either a C-shaped stylet or a double C-shaped stylet.

The limitation of this study is that it was conducted using a model, so the results may differ from clinical results. In addition, we used one videolaryngoscope (McGrath® MAC), and thus it is not clear if the results can be applicable to other types of videolaryngoscopes, because the efficacies are likely to be different between different videolaryngoscopes [4, 16]. Another possible limitation of the study is that the participants consisted of a mixture of specialists and residents, and each participant’s former experience of the use or non-use of a stylet with a different shape in daily clinical practice might have affected intubation time for each intubation method in this simulation study. Nevertheless, some participants usually would not use a stylet, whereas the others would usually use a C-shaped, J-shaped, or hockey stick-shaped stylet for tracheal intubation in anesthetized neonates, and thus there was no strong bias toward one method or two between these participants. Therefore, the differences in familiarity of intubation methods among these participants in daily clinical practice would not have strongly affected the results in this simulation study.

In conclusion, we have shown that, while time to tracheal intubation would be similar with and without the use of a stylet, the shape of the stylet would affect intubation time. The use of the J-shaped stylet or the hockey stick-shaped stylet may significantly make tracheal intubation more difficult. From these results, it would be necessary to compare the efficacy of the use of a C-shaped stylet or double C-shaped stylet with no use of a stylet in the ease of tracheal intubation using a McGrath® MAC videolaryngoscope in neonates.

## Data Availability

The data are available from the corresponding author on reasonable requests.
